# Supplementary material for: Transcriptome Analysis Reveals Vimentin-Induced Disruption of Cell–Cell Associations Augments Breast Cancer Cell Migration
Source: Cells. 2022 Dec 13;11(24):4035. doi: 10.3390/cells11244035 (PMC9776984; doi:10.3390/cells11244035)
Supplement: Supplementary file 1 [file cells-11-04035-s001.zip › DEGs and enrichment analysis.zip!/DEGs and enrichment analysis/WT_VIMvsVector KEGG/ALL/WT_VIMvsVector_ALL.pathway_map.zip!/WT_VIMvsVector_ALL.pathway_map/src/hsa05146.html]

hsa05146


- hsa:3918

- Down regulated genes

ENSG00000058085(-2.27781883943368)
- hsa:3915

- Down regulated genes

ENSG00000135862(-1.63927617256978)
- hsa:3909

- Down regulated genes

ENSG00000053747(-1.74718362506906)

- hsa:3576

- Down regulated genes

ENSG00000169429(-3.18264312774765)

- hsa:7097

- Down regulated genes

ENSG00000137462(-2.15431223286635)

- hsa:7097

- Down regulated genes

ENSG00000137462(-2.15431223286635)

- hsa:107

- Up regulated genes

ENSG00000164742(1.87205666251591)

- hsa:7042

- Down regulated genes

ENSG00000092969(-2.32734226266555)

- hsa:1287

- Down regulated genes

ENSG00000188153(-2.16664393151527)
- hsa:1285

- Down regulated genes

ENSG00000169031(-2.54975234049394)

- hsa:1287

- Down regulated genes

ENSG00000188153(-2.16664393151527)
- hsa:1285

- Down regulated genes

ENSG00000169031(-2.54975234049394)
- hsa:1281

- Up regulated genes

ENSG00000168542(1.50071136748401)

Close
